# Berberine Ameliorates Doxorubicin-Induced Cardiotoxicity via a SIRT1/p66Shc-Mediated Pathway

**DOI:** 10.1155/2019/2150394

**Published:** 2019-12-06

**Authors:** Yan-Zhao Wu, Lan Zhang, Zi-Xiao Wu, Tong-tong Shan, Chen Xiong

**Affiliations:** ^1^Department of Otorhinolaryngology-Head and Neck Surgery, 4th Hospital of Hebei Medical University, Shijiazhuang 050011, China; ^2^Basic Medical College, Chengde Medical College, Chengde 067000, Hebei Province, China; ^3^The Key Laboratory of Neural and Vascular Biology, Ministry of Education, Hebei Medical University, Shijiazhuang 050017, China; ^4^The Key Laboratory of Pharmacology and Toxicology for New Drugs, Department of Pharmacology, Hebei Medical University, Shijiazhuang 050017, China

## Abstract

Doxorubicin- (DOX-) induced cardiotoxicity is associated with oxidative stress and cardiomyocyte apoptosis. The adaptor protein p66Shc regulates the cellular redox status and determines cell susceptibility to apoptosis. This study is aimed at investigating the involvement of sirtuin 1- (SIRT1-) mediated p66Shc inhibition in DOX-induced redox signalling and exploring the possible protective mechanisms of berberine (Ber) against DOX-triggered cardiac injury in rats and a cultured H9c2 cell line. Our results showed that the Ber pretreatment markedly increased CAT, SOD, and GSH-PX activities, decreased the levels of MDA, and improved the electrocardiogram and histopathological changes in the myocardium in DOX-treated rats (in vivo). Furthermore, Ber significantly ameliorated the DOX-induced oxidative insult and mitochondrial damage by adjusting the levels of intracellular ROS, ΔΨ_m_, and [Ca^2+^]_m_ in H9c2 cells (in vitro). Importantly, the Ber pretreatment increased SIRT1 expression following DOX exposure but downregulated p66Shc. Consistent with the results demonstrating the SIRT1-mediated inhibition of p66Shc expression, the Ber pretreatment inhibited DOX-triggered cardiomyocyte apoptosis and mitochondrial dysfunction. After exposing H9c2 cells to DOX, the increased SIRT1 expression induced by Ber was abrogated by a SIRT1-specific inhibitor (EX527) or the use of siRNA against SIRT1. Accordingly, SIRT1 inhibition significantly abrogated the suppression of p66Shc expression and protection of Ber against DOX-induced oxidative stress and apoptosis. These results suggest that Ber protects the heart from DOX injury through SIRT1-mediated p66Shc suppression, offering a novel mechanism responsible for the protection of Ber against DOX-induced cardiomyopathy.

## 1. Introduction

Doxorubicin (DOX) is a first-line anthracycline quinone and an effective anticancer drug extensively used in clinical practice [1]. However, the clinical use of DOX is limited due to its dose-dependent and cumulative cardiotoxicity, which may cause dilated cardiomyopathy and heart failure [2, 3]. Multiple mechanisms are involved in DOX-induced cardiotoxicity, including an increase in reactive oxygen species (ROS) and lipid peroxidation, calcium overloading, and deterioration of mitochondrial function, leading to impaired DNA and cardiomyocyte apoptosis [4, 5]. In particular, growing evidence suggests that the overproduction of reactive oxygen species (ROS) and increased oxidant-induced mitochondrial damage are highly important for the development of the cardiotoxic effect of DOX [1, 6]. Consequently, multiple cardioprotective treatment strategies have been proposed as potential solutions. Using antioxidants could be an important strategy for partially protecting cardiac cells from DOX-induced oxidative damage and cardiotoxicity. Nevertheless, antioxidant supplementation achieved minimal success even though these attempts produced beneficial effects [7, 8]. These studies indicate that other mechanisms may be involved in DOX-induced cardiotoxicity. Recent studies conducted by Sampaio et al. demonstrated that the toxicity associated with DOX is mediated by p66Shc signalling [9], but the underlying mechanism has not been fully elucidated. Therefore, exploring novel therapeutic strategies for preventing DOX-induced cardiotoxicity without reducing its anticancer efficacy remains a major challenge.

The 66 kDa Src homology 2 domain-containing protein (p66Shc) is a newly recognized intracellular critical mediator that converts oxidative signals into ROS and has been implicated in promoting mitochondrial oxidative signalling into apoptosis [10, 11]. Rodents with a genetic knockout of p66Shc demonstrate a lifespan that is approximately 30% longer and significant resistance to oxidative stress and oxidative stress-dependent pathologies [12–14]. Recent studies have demonstrated that the expression of p66Shc is significantly upregulated in DOX-induced cardiotoxicity [10, 15]. Hence, the selective inhibition of the p66shc pathway is a promising approach for circumventing DOX cardiotoxicity. As a negative regulator of oxidative stress, silent mating type information regulation 2 homolog 1 (SIRT1) functions as a nicotinamide adenine dinucleotide- (NAD+-) dependent class III histone deacetylate (HDAC) [16, 17]. A more recent study illustrated that p66Shc is indeed a target of SIRT1 and that its expression can be at least partially decreased through SIRT1 upregulation [18, 19], while the knockdown of SIRT1 increased p66Shc expression [20]. Therefore, we propose that the SIRT1-mediated inhibition of p66Shc may be involved in DOX-induced cardiotoxicity, but unfortunately, the exact roles of the SIRT1-p66shc pathway remain controversial. To determine whether the SIRT1-p66shc pathway is involved in this process, berberine (Ber), an antioxidant, was administered to rats, and its effects were also assessed using in vitro cell culture studies.

Ber, which is a type of alkaloid, was originally extracted from a Chinese plant and has been used as a broad-spectrum antibiotic [21]. Ber has many pharmaceutical characteristics, such as antitumour and cardioprotective actions [22, 23]. Increasing clinical trial studies have revealed the good protecting effects of Ber on metabolic syndrome and cardiovascular diseases [24–26]. Interestingly, several studies have reported that Ber may confer a protective effect by activating SIRT1 signalling under various pathological conditions [27, 28]. However, whether SIRT1 signalling participates in the protective effect of Ber in DOX-induced cardiac dysfunction and its underlying mechanisms remains undefined. Therefore, the aims of the present study were to (1) examine the protective ability of Ber against DOX-induced cardiotoxicity by regulating ROS generation, mitochondrial damage, and apoptosis; (2) determine whether the SIRT1/p66Shc pathway is involved in DOX-induced cardiotoxicity in rats and H9c2 cells; and (3) determine whether the regulating protective effect of Ber is mainly related to the modulation of the SIRT1/p66Shc pathway.

## 2. Materials and Methods

### 2.1. Reagents

DOX was provided by Lingnan Pharmaceutical, Ltd., China. Ber was provided by Acros Organics, Belgium. EX-527 were purchased from Sigma Chemical Co. (St. Louis, MO, USA). Rhodamine (Rh-123) and rhod-2-AM were purchased from Molecular Probes (Eugene, OR, USA). The malondialdehyde (MDA), catalase (CAT), lactate dehydrogenase (LDH), glutathione peroxidase (GSH-PX), and superoxide dismutase (SOD) assay kits were purchased from Nanjing Jiancheng Bioengineering Institute (Nanjing, China). 2′,7′-Dichlorofluorescin diacetate (DCFH-DA) was purchased from Beyotime institute of Biotechnology. All other chemicals and regents were purchased from local agencies. The primary antibodies against SIRT1, MnSOD (manganese superoxide dismutase), cleaved caspase-3, Bcl-xL (B-cell lymphoma extra large), and *β*-actin and SIRT1 siRNA were purchased from Santa Cruz Biotechnology Co. (Santa Cruz, CA, USA).

### 2.2. Animals and Treatments

Sprague-Dawley rats weighing 220-250 g were obtained from the medical laboratory of Hebei Medical University. All animals were housed in the new environment for one week in a standard experimental room (12 h light/dark cycle) with free access to tap water. This study conformed to the Guide for the Care and Use of Laboratory Animals (NIH Publication No. 85-23, Revised 2016). All experimental procedures performed in the current study followed the guidelines of the research ethics committee of Hebei Medical University (Shijiazhuang, China). In the current study, forty rats were randomly separated into the following four groups: control (0.9% saline), DOX treatment (20 mg/kg), and DOX plus Ber treatment (10 and 20 mg/kg). The control and DOX groups received equal volumes of 0.9% saline intragastrically for 10 days. Ber was orally administered at the previously mentioned doses once daily for 10 days. Then, all rats, except for the rats in the control group, received intraperitoneal injections of DOX (20 mg/kg/day diluted with 0.9% saline) every other day for a total of three injections. Nine days after the first administration of DOX, the rats were euthanized for the subsequent studies.

### 2.3. Histopathological Examinations

To analyse the histopathologic changes in cardiac tissue, the rats were sacrificed with an intraperitoneal injection of 2 mL of pentobarbital. The heart was excised, and one part of the myocardium was fixed overnight in 10% formalin, embedded in paraffin, and dehydrated in an ascending series of ethanol (70, 80, 96, and 100%). The tissue samples were embedded in paraffin wax and cut into 5 *μ*m thick sections. The sections were mounted on normal glass slides and stained with haematoxylin and eosin (H&E) for 2 min for the histological examination. The specimens were examined under a light microscope (magnification, ×200, Olympus BX-50; Olympus Corporation, Tokyo, Japan).

### 2.4. Electrocardiography (ECG)

The rats were anesthetized and fixed on a table in the supine position using a Bioscience ECG recorder (Bioscience, Washington, USA). Subcutaneous needle electrodes were connected to the rats for the limb lead at position II, and electrocardiograms were recorded.

### 2.5. Cell Culture and Viability Analysis

The rat cardiac H9c2 cell line was obtained from the Chinese Academy of Sciences (Shanghai, China). The cells were cultured in DMEM medium supplemented with 1.5 g/L sodium bicarbonate, 10% FBS, 100 U/mL penicillin, and 100 *μ*g/mL streptomycin in tissue culture flasks at 37°C in a humidified atmosphere of 5% CO_2_. The H9c2 cells were treated with Ber at concentrations of 0.1, 1, or 10 *μ*M for 24 h and then exposed to 1 *μ*M DOX for 24 h to generate the cell injury model. In separate experiments, the cells were preincubated with 10 *μ*M EX-527 60 min before the addition of Ber. The same volumes of corresponding solvents were added to the controls. The cell viability was determined using a 3-(4,5-dimethylthiazol-2-yl)-2,5-diphenyl tetrazolium bromide (MTT) assay (Beyotime, Beijing, China). Briefly, the cells (1 × 10^5^/*mL*) were seeded in a 96-well culture plate and treated with different concentrations of Ber or DOX. After the treatment, 10 *μ*L MTT dye (5 mg/mL) was added to each well, and the cells were cultured for another 4 h of incubation. Then, the medium was discarded, and 150 *μ*L dimethyl sulfoxide (DMSO) was added to each well to dissolve the formazan crystals. The absorbance was measured using a microtiter plate reader (SpectraMax 190, Molecular Device, USA) at a wavelength of 490 nm.

### 2.6. Biochemical Index Analysis

The H9c2 cells were harvested, ultrasonicated, and centrifuged at 1000 rpm at 4°C for 5 min; then, the levels of SOD and MDA in the supernatant were detected using the corresponding detection kits. The culture medium was collected for the measurement of the lactate LDH release level using an LDH assay kit. In addition, the fresh heart tissue was rinsed, placed in cold saline (1 : 10, *w*/*v*), and homogenized using a homogenizer (T 18 basic Ultra-Turrax®; Mandel Scientific Company Inc., Guelph, Canada). Subsequently, the homogenate was centrifuged at 1000 × g for 15 min at 4°C for the detection of the MDA, SOD, CAT, and GSH-PX levels in the heart tissues. The supernatants were removed and used for a Western blot analysis.

### 2.7. Detection of Intracellular Reactive Oxygen Species (ROS)

To measure intracellular ROS formation, the fluorescent probe DCFH-DA was used. H9c2 cells were treated with DOX and Ber for specified time periods and attached to plates loaded with 10 *μ*mol/L DCFH-DA in the dark at room temperature for 20 min. Then, the cells were digested with trypsin, washed three times with PBS buffer, transferred to flow cytometer tubes, and recorded under a confocal scanning laser microscope (Becton Dickinson, USA) at an emission wavelength of 525 nm and an excitation wavelength of 488 nm. Finally, the ROS fluorescence in cardiomyocytes was analysed with PathVision imaging software.

### 2.8. Measurement of Mitochondrial Membrane Potential (ΔΨ_m_) and Mitochondrial Ca^2+^ Concentration ([Ca^2+^]_m_)

H9c2 cells were treated with DOX and Ber for specified time periods and incubated with 5 *μ*M Rh-123 (Molecular Probes, USA) for 30 min in the dark at 37°C. The cells were digested with trypsin, washed with warmed PBS twice, transferred to flow cytometer tubes, and detected with a flow cytometer (Becton Dickinson, USA) at an emission wavelength of 538 nm and an excitation wavelength of 485 nm. The mitochondrial Ca^2+^ concentration ([Ca^2+^]_m_) was measured using the Ca^2+^-fluorescent dye rhod-2-AM (Molecular Probes). Briefly, the H9c2 cultures were transferred to 1 mL fresh DMEM containing 10 *μ*M rhod-2 acetoxymethyl ester for 120 min at 4°C and then incubated for 30 min at 37°C. Then, the rhod-2-loaded cells were placed on the stage of a confocal microscope (Olympus), and emission was monitored through a 510 and 570 nm bandpass barrier filter to analyse [Ca^2+^]_m_. Finally, ΔΨ_m_ and [Ca^2+^]_m_ were calculated using the median fluorescence intensity of 10000 cells.

### 2.9. RNA Interference

The SIRT1 siRNA sequences were as follows: sense 5′-CCCUGUAAAGCUUUCAGAAdtdt-3′ and antisense 5′–UUCUGAAAGCUUUACAGGGdtdt-3′. The SIRT1-specific siRNA and control siRNA were obtained from Santa Cruz Biotechnology Co. (Santa Cruz, CA, USA), and transient siRNA transfection was carried out according to the manufacturer's instructions. After siRNA transfection for 48 h, a treatment with 1 *μ*M Ber was applied for 8 h. Finally, the proteins were extracted from the cells for Western blotting.

### 2.10. Western Blotting

To detect SIRT1, p66shc, MnSOD, Bcl-xL, cleaved caspase-3, and *β*-actin, the proteins were extracted from the myocardium or cultured cardiomyocytes, and equal amounts of protein (50 *μ*g) from each sample were separated by SDS-PAGE (Bio-Rad, Hercules, USA) and transferred to PVDF membranes. The membranes were blocked for 1 hr at 37°C with 5% nonfat dry milk in TBST (50 mM Tris-HCl, 150 mM NaCl, 0.1% Tween, pH 7.4) and incubated with the following antibodies overnight at 4°C: SIRT1, p66shc, MnSOD, Bcl-xL, cleaved caspase-3, and *β*-actin (1 : 500 in TBST). After washing with TBST three times, the samples were incubated with a secondary antibody in TBST solution for 30 min at 37°C. The proteins were exposed to an enhanced chemiluminescence plus system from the Bioengineering Institute of Biotechnology (Beijing, China). The Western blot signals were scanned and quantified by a densitometric analysis. The emitted light was captured using a BioSpectrum-410 multispectral imaging system and analysed using a Gel-Pro Analyzer (Version 4.0; Media Cybernetics, Rockville, MD). The protein expression levels were normalised to the ratios of *β*-actin detected on the same blot to control for the relative signal intensity.

### 2.11. Statistical Analyses

The data analysis was performed by using Social Science 11.0 (SPSS/PC), and the data are expressed as the *means* ± *standard* *deviation* (*SD*) of the experiments as indicated in the figure legends. Multiple comparisons were performed using a one-way analysis of variance (ANOVA), followed by Bonferroni multiple comparison post hoc tests. Comparisons between two groups were performed by using Student's *t*-test. Significance was accepted at *P* values < 0.05.

## 3. Results

### 3.1. Protective Effect of Ber against Acute DOX-Induced Cardiac Injury

**Figure 1:**
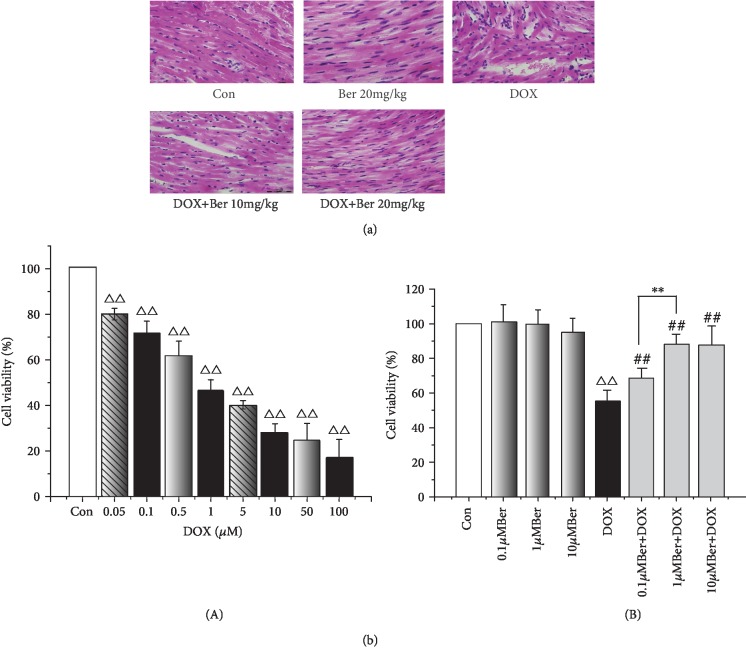
Ber diminishes DOX-induced cardiac injury. Rats were treated with Ber before the administration of DOX. (a) Effects of Ber on histopathological changes in DOX-treated cardiac tissue by H&E staining (magnification, ×200). Con: normal cardiac tissue; Ber: berberine (20 mg/kg); DOX: doxorubicin (20 mg/kg); DOX+Ber 10 mg/kg, and DOX+Ber 20 mg/kg. (b) Impact of DOX and Ber on cell viability. H9c2 cells were pretreated with Ber (0.1, 1, and 10 *μ*M) for 24 h and exposed to 1 *μ*M DOX for 24 h; cell viability was determined by an MTT assay and is expressed as a percentage relative to the control group. (A) Dose-dependent effect of DOX on cell viability in H9c2 cells; (B) effects of Ber on the reduction in the viability rate induced by DOX in H9c2 cells. The results are presented as the mean ± SD (*n* = 6). ^△△^*P* < 0.01 vs. control group, ^##^*P* < 0.01 vs. DOX group, and ^∗∗^*P* < 0.01 vs. Ber+DOX group.

We first explored whether Ber could protect rats from acute DOX-induced cardiac injury. Three rats in the DOX-only-treated group (33.3%) died two days after the DOX administration. However, no mortality was observed in any of the other groups, including the combined Ber+DOX-treated group. The influence of the treatment with Ber and/or DOX on the rat ECG parameters is shown in Table 1. The ECG tracing showed no changes in the heart rate in all experimental groups; the rats in the control and Ber (10 mg/kg) groups had a mean heart rate of 452.0 ± 50.0 and 434.0 ± 68.0 beats/min, respectively. The rats in the DOX-only-treated group showed several ECG changes, including bradycardia (381.0 ± 41.0 beats/min), and the prolongation of both ST and QRS complexes and QT intervals in the DOX-only-treated group was statistically significant compared to that in the control group (*P* < 0.05 or *P* < 0.01). Such ECG abnormalities were obviously improved in the Ber+DOX group as evidenced by the normalization of the heart rate, ST, and both QRS complexes and QT intervals (Table 1). The protective effect of Ber was further verified by a histopathological analysis (Figure 1(a)). The tissues from the rats exposed to DOX revealed myocardial cell injury, including capillary congestion, interstitial oedema, and inflammation infusion, in different degrees. In contrast, the Ber pretreatment dramatically reduced the DOX-induced histopathological damage. Subsequently, the cytoprotective effect of Ber was then examined in vitro using an H9c2 rat cardiomyocyte cell model. To determine the cell viability following treatment with different concentrations of DOX and Ber in the cell line, an MTT assay was performed. The exposure of the H9c2 cells to DOX for 24 h resulted in a significant reduction in cell viability in a dose-dependent manner (Figure 1(b), A). The LC_50_ based on the dose-response curves was 1.02 *μ*M for the 24 h time point. Based on this result, we used 1 *μ*M DOX for 24 h as the treatment in this work. However, the pretreatment of the cells with Ber attenuated the DOX effect on cell viability in a dose-dependent manner. As shown in Figure 1(b), B, 0.1 *μ*M and 1 *μ*M Ber significantly increased cell survival by 29% and 43%, respectively, compared with that in the DOX-treated cells (*P* < 0.01). A higher concentration of Ber showed no additional benefit to cell viability. Therefore, the 1 *μ*M dose of Ber was selected for the subsequent in vitro experiments.

### 3.2. Ber Reduced DOX-Induced Oxidative Stress In Vivo and In Vitro

**Figure 2:**
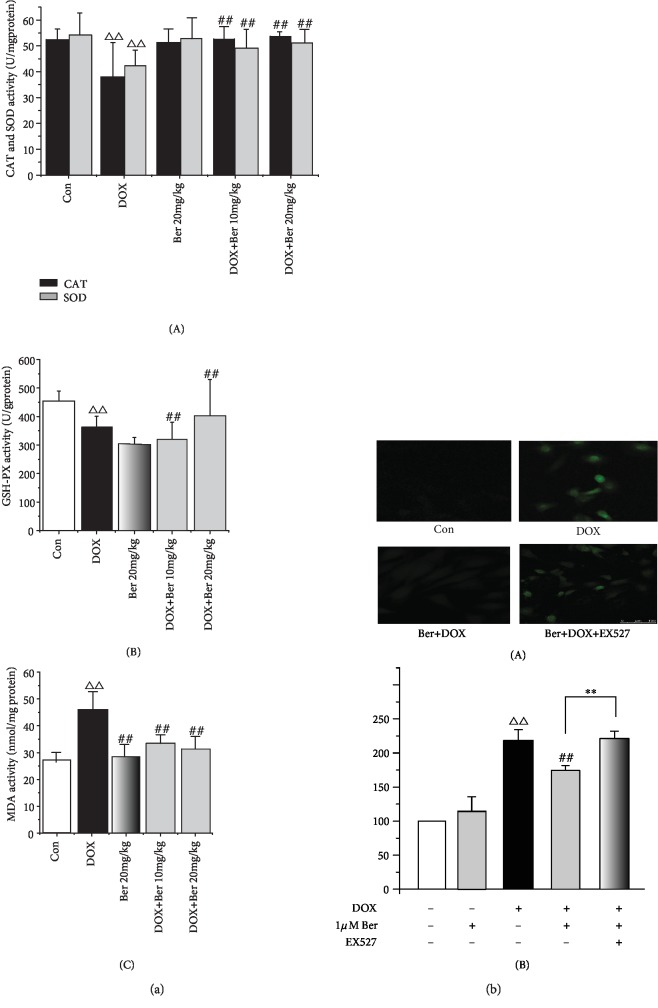
Ber ameliorated DOX-induced oxidative stress in vivo and in vitro. (a) Ber improves cardiac enzyme antioxidative activity: (A) cardiac CAT and SOD levels in rats; (B) cardiac GSH-PX level in rats; (C) cardiac MDA level in rats. (b) Effects of Ber on the increase in ROS level induced by DOX in H9c2 cells. Intracellular ROS production was detected using flow cytometry: (A) representative flow cytometry histograms; (B) quantitative analysis of (a). The results are presented as the mean ± SD (*n* = 15). ^△△^*P* < 0.01 vs. control group, ^##^*P* < 0.01 vs. DOX group, and ^∗∗^*P* < 0.01 vs. Ber+DOX group.

CAT, SOD, MDA, and GSH-PX are major markers of oxidative stress and are regarded as indicators of oxidative injury [29, 30]. Since oxidative stress is considered the primary cause of DOX-induced cardiomyopathy, we further measured the levels of these markers to investigate the effects of Ber on myocardial superoxide generation induced by DOX. As shown in Figure 2(a), the cardiac activities of CAT, SOD, and GSH-PX were significantly reduced by the DOX alone treatment compared to those in the controls, while the MDA levels were increased (*P* < 0.01). Meanwhile, the activities of CAT, SOD, and GSH-PX in the combined Ber+DOX group were significantly elevated compared to those in the DOX treatment alone group. In contrast, the DOX-induced elevation in the MDA levels was markedly reduced in the Ber-treated groups (*P* < 0.01). Next, we measured the cytotoxicity of DOX in H9c2 cardiomyoblasts by using the DCFH technique. Compared to the normal control, the treatment with 1 *μ*M DOX for 24 h significantly increased the production of ROS in the H9c2 cells from 100% to 221.7 ± 2.9% (*P* < 0.01) (Figure 2(b)). The pretreatment with 1 *μ*M Ber for 24 h dramatically attenuated the production of ROS by 23.2% (170.1 ± 11.76%, *P* < 0.01) compared with that in the DOX-treated cells. However, this protective effect of Ber was reversed by the addition of the SIRT1 inhibitor EX527 60 min prior to the exposure of the cells to Ber plus DOX (*P* < 0.01).

### 3.3. Ber-Mediated Protection against DOX Involves SIRT1/p66Shc Activation

**Figure 3:**
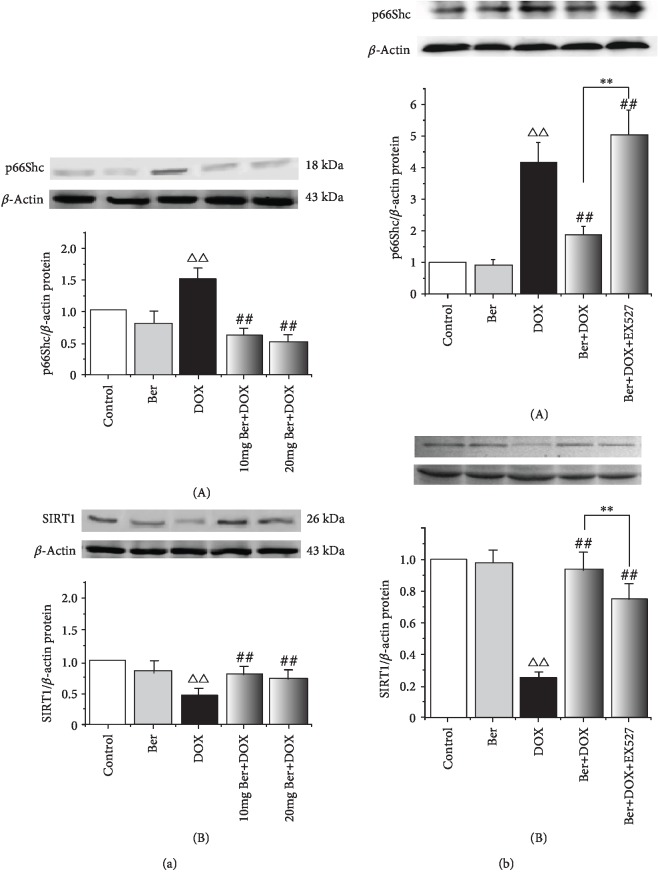
Effects of Ber on p66Shc and SIRT1 expression in vivo and in vitro as analysed by a Western blot analysis and quantified by a densitometric analysis. (a) Western blot analysis of cardiac p66Shc and SIRT1 protein levels in rats: (A) representative blots of p66Shc expression; (B) representative blots of SIRT1 expression. ^△△^*P* < 0.01 vs. control group, ^##^*P* < 0.01 vs. DOX group. (b) Western blot analysis of cardiac p66Shc and SIRT1 protein levels in H9c2 cells treated with DOX, Ber+DOX, or Ber+DOX+EX527. H9c2 cells were treated with or without 10 *μ*M EX527 for 60 min, exposed to 1 *μ*M Ber for 24 h, and then exposed to 1 *μ*M DOX for 24 h: (A) representative blots of p66Shc expression; (B) representative blots of SIRT1 expression. The results are presented as the mean ± SD (*n* = 8). ^△△^*P* < 0.01 vs. control group, ^##^*P* < 0.01 vs. DOX group, and ^∗∗^*P* < 0.01 vs. Ber+DOX group.

To test the hypothesis that p66shc downregulation and SIRT1 activation are associated with the Ber-mediated attenuation of DOX, we measured the changes in the expression of SIRT1 and p66shc in response to Ber in vivo and in vitro. As shown in Figure 3(a), the results of the Western blot assay showed that p66shc expression was increased in the myocardium from the DOX-treated rats and that this increase was dramatically reversed after the Ber intervention in a dose-dependent manner; in contrast, SIRT1 expression was decreased in the myocardium from the DOX-treated rats compared with that in the control group. However, Ber abrogated the decrease in SIRT1 protein expression, suggesting SIRT1/p66shc pathway activation. Then, we investigated whether the in vitro results could be recapitulated in H9c2 cells. In accordance with the in vivo data, p66shc protein expression was markedly increased in the H9c2 cells treated with 1 *μ*M DOX, and an obvious decrease in SIRT1 expression was observed (*P* < 0.01) (Figure 3(b)). In contrast, Ber increased SIRT1 expression but significantly inhibited p66shc expression. We hypothesized that the protective effects of Ber in DOX-induced cardiotoxicity may be related to the SIRT1-mediated inhibition of p66shc expression. To further test the above hypothesis, we pretreated H9c2 cells with the SIRT1 inhibitor EX527 for 1 h before the 24 h 1 *μ*M DOX treatment. In agreement with a previous observation showing that SIRT1 negatively regulates p66shc expression [20], our data further show that compared with the combined DOX+Ber group, the Ber-induced downregulation of p66shc and upregulation of SIRT1 were significantly attenuated by EX527 (Figure 3(b)).

### 3.4. SIRT1 Activation Protects Cardiomyocytes from DOX-Induced Mitochondrial Injury

**Figure 4:**
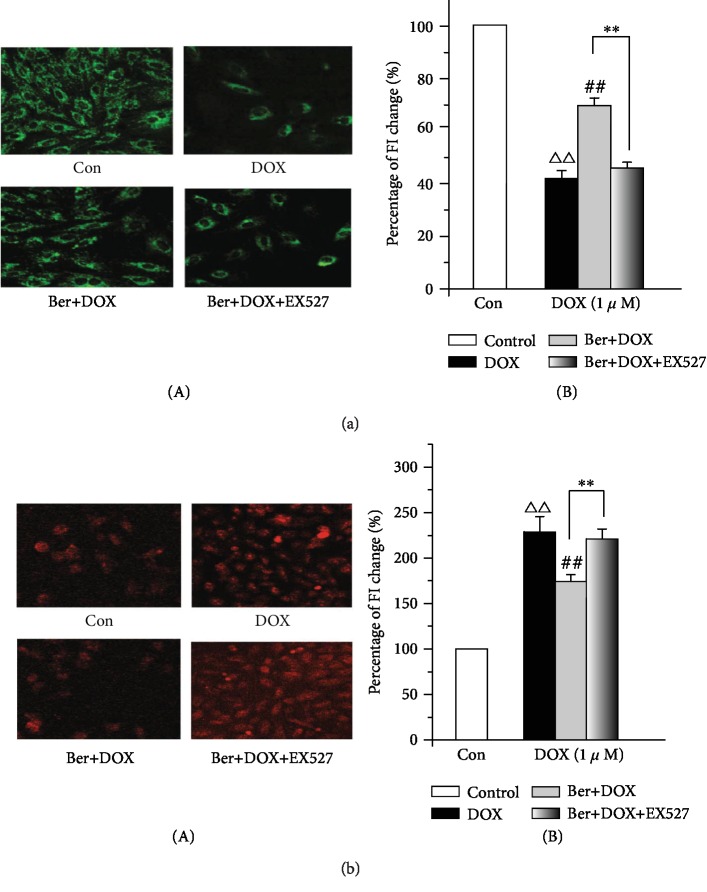
DOX-induced mitochondrial dysfunction can be significantly attenuated by Ber. (a) Effect of Ber on the decrease in the mitochondrial membrane potential (MMP) induced by DOX in H9c2 cells: (A) confocal images of ΔΨ_m_ in H9c2 cells (magnification, ×40); (B) summary data of the relative changes in Rh-123 fluorescence. (b) Effect of Ber on mitochondrial Ca^2+^ ([Ca^2+^]_m_) overload in rhod-2-AM-loaded H9c2 cells: (A) confocal images of [Ca^2+^]_m_ in H9c2 cells (magnification, ×40); (B) summary data of the relative changes in Rhod 2-AM fluorescence. The results are presented as the mean ± SD. The data are representative of three separate experiments, each with three technical replicates (*n* = 9). ^△△^*P* < 0.01 vs. control group, ^##^*P* < 0.01 vs. DOX group, and ^∗∗^*P* < 0.01 vs. Ber+DOX group.

Mitochondria are the target organelles of DOX toxicity in cardiomyocytes [5]. Mitochondria play an important role in the maintenance of Ca^2+^ homeostasis primarily because of their capacity to buffer cytosolic Ca^2+^ [31, 32]. ΔΨ_m_ is the central parameter that controls the accumulation of Ca^2+^ within the mitochondrial matrix, cell respiration, and ATP synthesis. Furthermore, Rhodamine 123 (Rh-123) was used to monitor ΔΨ_m_ in H9c2 cardiomyocytes to evaluate mitochondrial damage. Next, we examined whether the inhibition of p66shc by SIRT1 protected against DOX-induced mitochondrial injury and whether the protective effect of Ber involved this pathway. The corresponding changes in the Rh-123 fluorescence intensity in the cardiomyocytes were measured after perfusion of 1 *μ*M DOX.  Figure 4(a), A shows confocal images of ΔΨ_m_ after the cell treatment with 1 *μ*M DOX. The results revealed that DOX induced a marked decrease in the mitochondrial membrane potential. However, the pretreatment of the myocytes with 1 *μ*M Ber significantly restored the DOX-induced reduction in ΔΨ_m_ (Figure 4(a), A) and attenuated the DOX-induced decrease in the Rh-123 fluorescence intensity (Figure 4(a), B). More importantly, the protective effect of Ber against DOX was abrogated by EX527. Thus, these results indicate that Ber prevented the DOX-induced loss of the mitochondrial membrane potential, which may be associated with SIRT1 activation. Because mitochondrial Ca^2+^ overload leads to mitochondrial dysfunction, we addressed the question of whether Ber could attenuate the increase in the mitochondrial calcium level caused by the DOX treatment. The Rhod-2 fluorescence was monitored to evaluate the mitochondrial Ca^2+^ handling properties. Similar results were observed for mitochondrial Ca^2+^ ([Ca^2+^]_m_); the cardiomyocytes in the DOX alone group displayed a significant increase in [Ca^2+^]_m_ compared with those in the control group, while the Ber pretreatment significantly negated the DOX-induced dysfunction of [Ca^2+^]_m_ (Figure 4(b)). In contrast, these alterations could be reversed by inhibiting SIRT1 with EX527. Our data indicate that the Ber treatment markedly prevented DOX-induced ΔΨ_m_ and [Ca^2+^]_m_ in cardiomyocytes, which have been considered indicators of mitochondrial damage in cardiac diseases [33].

### 3.5. SIRT1-Mediated Inhibition of p66shc Expression Protects against Dox-Induced Oxidative Stress and Apoptosis

**Figure 5:**
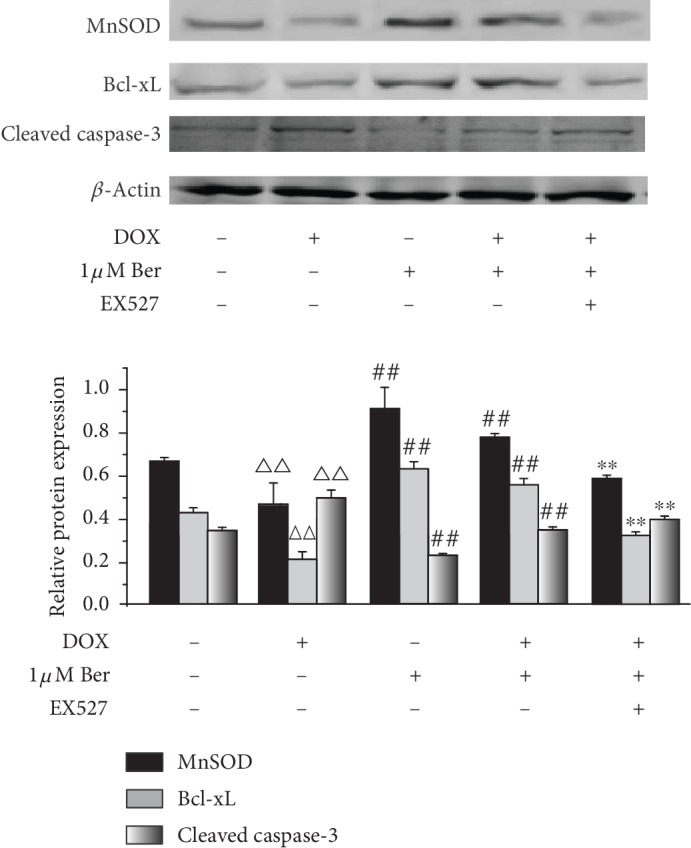
Sirtuin 1 (SIRT1) inhibition of p66shc protects against DOX-induced oxidative stress and apoptosis in H9c2 cells. Expression of manganese superoxide dismutase (MnSOD), Bcl-xL, and cleaved caspase-3 proteins was measured by Western blotting using specific antibodies. The data are presented as the means ± SD (*n* = 3). ^△△^*P* < 0.01 vs. control group, ^##^*P* < 0.01 vs. DOX group, and ^∗∗^*P* < 0.01 vs. Ber+DOX group.

It is known that DOX induces a pathophysiological mechanism characterized by increased ROS production and cardiomyocyte apoptosis [4, 34]. Therefore, we measured the effects of DOX and Ber on these indicators in vitro. To further explore the effect of the SIRT1/p66shc pathway of Ber on DOX-injured myocardial cells, we subsequently investigated the effect of Ber on the expression of oxidation- and apoptosis-related proteins, such as the superoxide scavenger MnSOD and apoptosis-related factors, including Bcl-xL and cleaved caspase-3. As shown in Figure 5, compared to the control group, the DOX administration enhanced the expression levels of cleaved caspase-3, which is a major marker of apoptosis, but downregulated MnSOD and Bcl-xL expression. Moreover, the pretreatment with Ber prior to the DOX exposure resulted in increased MnSOD and Bcl-xL accumulation but decreased cleaved caspase-3. These results suggest that the pretreatment with Ber inhibited DOX-induced oxidation and apoptosis in the H9c2 cells. To determine whether the antioxidant and antiapoptotic activity of Ber is associated with SIRT1, H9c2 cells were pretreated with the specific SIRT1 inhibitor EX725, and the pretreated cells were exposed to DOX; all cardioprotective effects of Ber were effectively abolished when SIRT1 was inhibited by EX527.

### 3.6. SIRT1 Deficiency Restricted the Protective Effects of Ber on DOX-Mediated Oxidative Stress Injury

**Figure 6:**
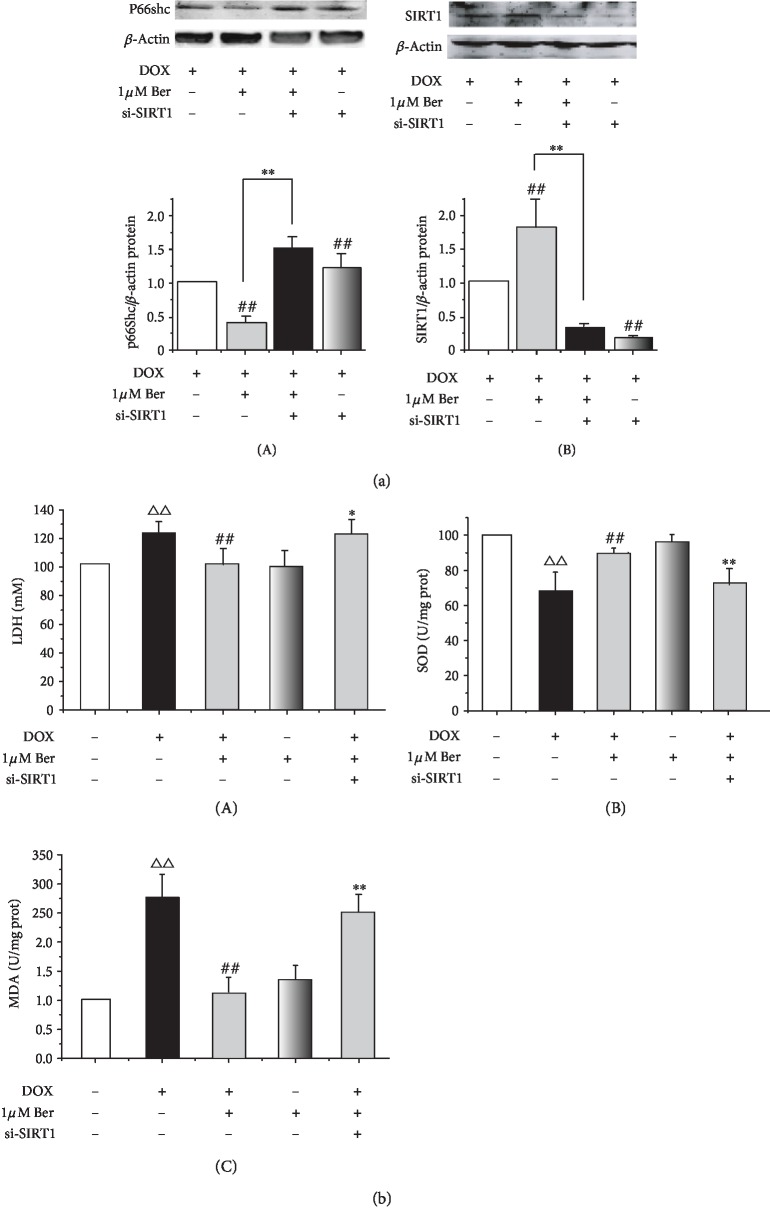
si-SIRT1 attenuated the protective effect of Ber in DOX-induced injured cardiomyocytes. H9c2 cardiomyocytes were exposed to control siRNA or SIRT1 siRNA and then treated with Ber (1 *μ*mol/L for 8 h). Then, the cells were exposed to 1 *μ*mol/L DOX for a 4 h treatment. Then, SIRT1-related signalling and oxidation-related proteins were measured. (a) Effects of Ber and SIRT1 siRNA on SIRT1 and p66Shc expression: (A) representative blots of p66Shc expression; (B) representative blots of SIRT1 expression. (b) si-SIRT1 restricted the antioxidative activity of Ber in DOX-stimulated H9c2 cells: (A) cardiac LDH level in rats; (B) cardiac SOD level in rats; (C) cardiac MDA level in rats. The results are expressed as the mean ± SD, *n* = 5‐8/group. ^△△^*P* < 0.01 vs. control siRNA group, ^##^*P* < 0.01 vs. DOX+control siRNA group, and ^∗^*P* < 0.05 or ^∗∗^*P* < 0.01 vs. DOX+Ber+control siRNA group.

To determine whether Ber confers cardioprotection in a SIRT1-dependent manner, we used the siRNA strategy to examine the role of SIRT1 in p66Shc regulation. Our results showed that compared with the siRNA control, the SIRT1 knockdown inhibited the effect of Ber in terms of the upregulation of SIRT1 in H9c2 cells. Furthermore, the Ber-induced downregulation of p66Shc was attenuated by the si-SIRT1-mediated SIRT1 silencing (Figure 6). In accordance with this result, as shown in Figure 6(b), DOX caused increases in the LDH and MDA activities in the H9c2 cardiomyocytes, while these changes were effectively ameliorated by the Ber and DOX combination compared to those observed in the cardiomyocytes treated with DOX alone (*P* < 0.01) (Figure 6(b), A and C). In contrast, the DOX-reduced SOD activity was markedly increased by the Ber combination compared to that observed with DOX alone (*P* < 0.01) (Figure 6(b), B). The SIRT1 knockdown by RNAi restricted the antioxidative defence activities of Ber.

## 4. Discussion

Doxorubicin (DOX) is a widely used anthracycline chemotherapeutic agent despite its cumulative and dose-dependent cardiotoxicity as a fatal side effect and subsequent development of cardiomyopathy and delayed clinical congestive failure [1–3]. The mechanism of DOX-induced cardiotoxicity has not been fully understood although it is still widely used. Additionally, it was recently shown that DOX activates the p66Shc pathway, leading to the translocation of the protein to the mitochondrial fraction and further generation of local oxidative stress [9, 15], which may contribute to uncovering a novel mechanism of DOX-induced cardiotoxicity. In this regard, p66Shc downregulation may hinder the development of DOX-induced toxicity.

In fact, under various pathological conditions and disease states, p66shc functions as an intracellular mediator that converts the mitochondrial route of apoptosis [35–37]. As an oxidative stress sensor, p66Shc translocated to mitochondrial membranes to increase ROS generation through its oxidoreductase activity and opening of the mitochondria permeability transition pore, which then contributes to organ dysfunction [38]. Additionally, mice with a genetic deletion of p66Shc display increased resistance to oxidative stress and apoptosis and are protected against tissue damage [38]. Our results confirmed that the p66Shc protein levels significantly increased in both the in vivo and in vitro models of DOX-induced cardiac damage (Figure 3). These findings suggest that p66shc plays an important role in the cardiotoxicity induced by DOX and may be a promising candidate for therapeutic intervention. However, the p66Shc signalling pathway is a complicated process, and no specific p66Shc inhibitors are currently available. Therefore, one attractive hypothesis suggests that upstream regulators of the p66Shc pathway may be potential candidates for combatting DOX injury. Growing experimental and population-based evidence suggests that SIRT1, which is a class III histone deacetylase, negatively regulates p66Shc expression through epigenetic chromatin modification [19]. Similarly, widespread evidence indicates that activated SIRT1 helps cellular resistance against oxidative stress [39–41]. Based on these findings, we hypothesized that p66Shc may be a target of SIRT1 in DOX-induced cardiotoxicity. Here, in our study, the results of the Western blot analysis revealed that SIRT1 protein expression was obviously decreased, while p66Shc was significantly increased in the rat myocardium post-DOX treatment, which could be reversed by Ber intervention. In addition, the in vitro experiment provided further evidence demonstrating that EX527, which is a specific SIRT1 inhibitor, attenuated the inhibition of p66Shc expression caused by Ber in H9c2 cells exposed to DOX treatment (Figure 3). Altogether, these data imply that the Ber-mediated inhibition of p66Shc occurs at least partially via the activation of SIRT1 expression during DOX-induced cardiotoxicity, indicating that Ber may be a potential activator of SIRT1 and may protect against DOX-induced cardiotoxicity through SIRT1 upregulation. This finding is consistent with recent studies showing that SIRT1 plays a protective role against DOX-induced mitochondrial biogenesis dysfunction and cardiomyocyte toxicity [42–45]. The regulation of p66Shc expression by SIRT1 is likely due to the mechanism described by Zhou et al. by which SIRT1 decreases acetylated histone H3 binding to the p66shc promoter region and represses p66shc activity [41]. The present work indicated that SIRT1/p66Shc is a key therapeutic target for the prevention of DOX-induced cardiotoxicity and that the activation of the SIRT1/p66Shc pathway is involved in the cardioprotective effect of Ber in DOX.

Berberine (Ber) is an alkaloid extract from the Coptis chinensis species. Ber has a long history of use for the treatment of diarrhoea in oriental medicine [22]. Increasing studies have revealed that Ber is an effective antioxidant and free radical scavenger that possesses a variety of pharmacological and biological activities [23, 46, 47]. Attractively, investigations have shown that Ber exerts anticancer activity and can be a potential multispectrum anticancer agent [48, 49], indicating that a treatment combining Ber with DOX does not interfere with the antitumour effect of DOX and significantly inhibits cancer cell proliferation [50, 51]. In the present study, we demonstrated that Ber treatment exhibits a significant protective effect on cardiac tissue in animal and in vitro cell culture studies of DOX-induced cardiac injury as determined by several measurements. The Ber treatment significantly alleviated the DOX-induced global redox injury in rats by increasing antioxidant enzymes, such as CAT, SOD, and GSH-PX, and significantly reducing the levels of heart MDA, which is an end product of lipid hydroperoxide. Similarly, the Ber therapy significantly attenuated the histopathological deteriorations and ECG abnormalities. In parallel with this effect, Ber could protect DOX-induced H9c2 cardiomyocytes by ablating the increased ROS production and improving the cell survival ability caused by DOX. Some evidence indicates that the enhanced ROS generation by DOX is associated with the collapse of the mitochondrial membrane potential, thereby releasing the proapoptotic molecule cytochrome c and subsequently activating caspase-3 [52], ultimately resulting in myocyte apoptosis [53]. In this study, as shown in our experiments, DOX caused mitochondrial Ca^2+^ overload, resulting in the depolarization of the mitochondrial membrane (Figure 4), which may alter cardiac metabolism and lead to cardiomyocyte death. The incubation with Ber significantly reduced the mitochondrial Ca^2+^ overload and elevated ΔΨ_m_. Moreover, the incubation of the H9c2 cells with EX-527 significantly blocked the Ber-mediated prevention of DOX-induced mitochondrial dysfunction. These results indicate that Ber inhibited DOX-induced mitochondrial function in a SIRT1-dependent manner. This conclusion is supported by several studies reporting that the cardioprotective action of Ber is associated with the regulation of SIRT1 signalling under some pathological conditions [27, 28, 54]. Since Ber activates SIRT1 activity and SIRT1 can also regulate p66Shc activity, it is tempting to speculate that the protective effects of Ber against the effects of DOX may be partially mediated by SIRT1 suppressing p66Shc expression. Interestingly, our experiment revealed that the Ber pretreatment increased the protein expression of SIRT1 and ameliorated the DOX-induced p66Shc protein expression, whereas the Ber-mediated downregulation of p66shc in the H9c2 cells was attenuated by the inhibition of SIRT1 expression by EX-527 and si-SIRT1 (Figures 3 and 6). These findings suggest that the Ber treatment led to SIRT1 upregulation, indicating that the protective effects of Ber against DOX-induced injury maybe related to the SIRT1-mediated inhibition of p66shc expression. To further investigate the above hypothesis, we examined the effect of Ber on antioxidative and antiapoptotic pathways associated with SIRT1. As shown in Figure 5, the pretreatment with Ber prior to the DOX exposure resulted in an obvious decrease in ROS generation and cardiac apoptosis by increasing antioxidants, such as MnSOD, and the expression of antiapoptotic proteins, such as Bcl-xL, but conversely attenuating the expression of cleaved caspase-3; these changes were abolished by the coadministration of the SIRT1 inhibitor EX-527. Altogether, these data suggest that p66Shc overexpression causes cardiac oxidative damage, which plays a key role in DOX-induced cardiotoxicity, and indicate that Ber exerts an antioxidative and antiapoptotic effect via SIRT1-mediated p66Shc inhibition. The in vitro experiments with si-SIRT1 provide further evidence supporting this argument. We separately transfected H9c2 cells with SIRT1 siRNA. Our data showed that transfecting H9c2 cells with SIRT1 siRNA significantly attenuated the effect of Ber on p66shc and SIRT1 expression compared to the control siRNA group (Figure 6(a)), indicating that SIRT1 played key roles in the Ber repression of p66Shc expression. Oxidative stress is a cornerstone of DOX-induced cardiotoxicity. Here, as shown in Figure 6(b), oxidative damage was observed in the DOX-treated cardiomyocytes as shown by the markedly increased levels of LDH and MDA, which are released from damaged myocytes and are sensitive indicators of cardiac injury. Cardiac toxicity induced by DOX is manifested by decreased activities of the antioxidant enzyme SOD. However, these effects were abrogated by Ber. In contrast, as expected, the SIRT1 siRNA treatment attenuated these antioxidative effects of Ber. These findings revealed that Ber enhances the SIRT1-mediated repression of p66Shc expression and confers antioxidative protection against DOX-induced cardiotoxicity. After observing that Ber greatly attenuated DOX-induced cytotoxicity, we determined that the mitochondrial dysfunction and cell apoptosis were dependent on SIRT1; thus, further studies are needed to investigate the precise mechanism by which Ber modulates SIRT1 expression.

In conclusion, the present study reports novel data demonstrating that Ber has a protective effect against DOX-induced cardiovascular injury, which is correlated with the upregulation of SIRT1 and the downregulation of p66shc expression, resulting in suppressed ROS production, cellular apoptosis, and mitochondrial damage to improve cardiac dysfunction. This natural product should be developed as a new potential candidate to prevent or reduce the cardiac side effects of anthracyclines in chemotherapy. Of course, the exact mechanisms and clinical applications of this natural product to impede the progression of DOX-induced cardiotoxicity require further study.

Thus, our data suggest that the SIRT1-mediated repression of p66Shc expression contributes to the prevention of DOX-induced cardiotoxicity, suggesting that the SIRT1-p66Shc pathway may represent an attractive target of Ber and other candidates of cardioprotective adjuvants for the prevention/treatment of DOX-induced cardiovascular injury.

## 5. Conclusion

Our in vivo and in vitro results showed that Ber preconditioning afforded protection against DOX-induced cardiotoxicity by reducing oxidative stress and mitochondrial dysfunction. Mechanistically, the protective effect involves the sirtuin 1-mediated inhibition of p66Shc, suggesting that this pathway is a novel potential therapeutic target for decreasing DOX toxicity.

## Figures and Tables

**Table 1:** Influence of berberine on ECG parameters of rats treated with doxorubicin (mean ± SD).

Group	*n*	ST interval (ms)	QRS (ms)	QT interval (ms)	Heart rate (beat/min)
Con	10	2.5 ± 1.0	23.8 ± 4.2	57.6 ± 6.3	452.0 ± 50.0
DOX	8	3.0 ± 0.8^△^	29.0 ± 5.3^△△^	69.8 ± 9.1^△^	381.0 ± 41.0^△△^
Ber 10 mg/kg	10	2.5 ± 1.1^#^	25.6 ± 8.1^#^	61.3 ± 9.5^#^	434.0 ± 68.0^#^
Ber 20 mg/kg	10	2.7 ± 1.3^#^	24.2 ± 7.8^#^	59.2 ± 11.6^#^	463.0 ± 48.0^##^

^△^ *P* < 0.05, ^△△^*P* < 0.01*vs.* Con; ^#^*P* < 0.05, *^##^P* < 0.01*vs.* DOX.

## Data Availability

The datasets used and/or analysed during the current study are available from the corresponding author upon reasonable request.

## Abbreviations

DOX: Doxorubicin
Ber: Berberine
ROS: Reactive oxygen radical species
ΔΨ_m_: Mitochondrial membrane potential
[Ca^2+^]_m_: Mitochondrial Ca^2+^
LDH: Lactate dehydrogenase
MDA: Malondialdehyde
SOD: Superoxide dismutase
GSH-PX: Glutathione peroxidase
CAT: Catalase
Bcl-xL: B-cell lymphoma extra large.
